# Stem cell-derived and circulating exosomal microRNAs as new potential tools for diabetic nephropathy management

**DOI:** 10.1186/s13287-021-02696-w

**Published:** 2022-01-24

**Authors:** Lei Peng, Yu Chen, Shaoqing Shi, Heling Wen

**Affiliations:** 1 Department of Nephrology, Sichuan Academy of Medical Science and Sichuan Provincial People’s Hospital, Chengdu, 610072 China; 2 Department of Cardiology, Sichuan Academy of Medical Science and Sichuan Provincial People’s Hospital, Chengdu, 610072 China; 3 Department of Pulmonary and Critical Care Medicine, The First Affiliated Hospital of Kunming Medical University, Kunming, 650032 China

**Keywords:** Biomarker, Exosome, Diabetic nephropathy, microRNA, Serum, Stem cell, Urine

## Abstract

**Background:**

Despite major advances in the treatment of diabetic nephropathy (DN) in recent years, it remains the most common cause of end-stage renal disease. An early diagnosis and therapy may slow down the DN progression. Numerous potential biomarkers are currently being researched. Circulating levels of the kidney-released exosomes and biological molecules, which reflect the DN pathology including glomerular and tubular dysfunction as well as mesangial expansion and fibrosis, have shown the potential for predicting the occurrence and progression of DN. Moreover, many experimental therapies are currently being investigated, including stem cell therapy and medications targeting inflammatory, oxidant, or pro-fibrotic pathways activated during the DN progression. The therapeutic potential of stem cells is partly depending on their secretory capacity, particularly exosomal microRNAs (Exo-miRs). In recent years, a growing line of research has shown the participation of Exo-miRs in the pathophysiological processes of DN, which may provide effective therapeutic and biomarker tools for DN treatment.

**Methods:**

A systematic literature search was performed in MEDLINE, Scopus, and Google Scholar to collect published findings regarding therapeutic stem cell-derived Exo-miRs for DN treatment as well as circulating Exo-miRs as potential DN-associated biomarkers.

**Findings:**

Glomerular mesangial cells and podocytes are the most important culprits in the pathogenesis of DN and, thus, can be considered valuable therapeutic targets. Preclinical investigations have shown that stem cell-derived exosomes can exert beneficial effects in DN by transferring renoprotective miRs to the injured mesangial cells and podocytes. Of note, renoprotective Exo-miR-125a secreted by adipose-derived mesenchymal stem cells can improve the injured mesangial cells, while renoprotective Exo-miRs secreted by adipose-derived stem cells (Exo-miR-486 and Exo-miR-215-5p), human urine‐derived stem cells (Exo-miR-16-5p), and bone marrow-derived mesenchymal stem cells (Exo-miR-let-7a) can improve the injured podocytes. On the other hand, clinical investigations have indicated that circulating Exo-miRs isolated from urine or serum hold great potential as promising biomarkers in DN.

## Key message

Stem cell-derived and circulating Exo-miRs provide valuable insights to identify the ideal candidates for improving the therapeutic and diagnostic/prognostic goals in diabetic patients who are at a high risk of DN progression.

## Background

### The pathophysiology of diabetic nephropathy

Diabetic nephropathy (DN) is the main reason for end-stage renal disease (ESRD) worldwide, presenting a leading cause of morbidity and mortality in patients with either type I DM (T1DM) or type II DM (T2DM) [1]. DN is mainly characterized by functional and morphological abnormalities within the kidney. Morphological abnormalities include glomerular hypertrophy, podocyte injury and depletion, progressive accumulation of extracellular matrix (ECM), expansion of the mesangial matrix, and thickening of glomerular basement membrane (GBM), as well as glomerulosclerosis and tubulointerstitial fibrosis. Functional abnormalities including proteinuria, the reduced rate of glomerular filtration, as well as glomerular hyperperfusion and hyperfiltration occur before the initiation of morphological abnormalities [2]. Long-lasting hyperglycemia, high blood pressure, and inflammation are the major etiological factors responsible for DN development. These factors contribute to progressive and irreversible injury to the renal glomeruli and tubulointerstitial, causing the regression of renal function and the eventual renal failure [1].

When experiencing high-pressure conditions, podocytes and mesangial cells release various mediators that promote the functional and morphological alterations in the glomeruli [3]. These mediators include vascular endothelial growth factor A (VEGFA), transforming growth factor-β1 (TGF-β1), glomerular capillary remodeling cytokine, angiotensin II (Ang II) and angiotensin-converting enzyme (ACE), as well as pro-inflammatory cytokines such as chemokine (C–C motif) ligand 2/ monocyte chemoattractant protein-1 (CCL2/MCP-1) and interleukin-6 (IL-6) [4]. These mediators promote pathogenic alterations either directly via activation of cellular remodeling signaling pathways resulting in cellular morphological alterations and increasing ECM synthesis or indirectly by elevating oxidative stress through activating nicotinamide adenine dinucleotide phosphate hydrogen (NADPH) oxidase [4].

Hyperglycemia produces advanced glycation end products (AGEs) within plasma and tissues. AGEs promote kidney complications through two independent pathways. On the one hand, AGEs irreversibly attach to matrix proteins (laminin and type IV collagen) and inhibit their degradation via matrix metalloproteinases, which puts up fibrosis by the excessive deposition of ECM proteins [5, 6]. On the other hand, AGEs also interact with their receptors expressed by podocytes and mesangial cells and, thereby, promote specific cellular responses including the secretion of pro-fibrotic cytokines, such as VEGF, connective tissue growth factor (CTGF), and TGF-β1, as well as elevated expression of NADPH oxidase. Altogether, these result in glomerular cell proliferation, expansion, or hypertrophy [4, 7].

Kidney inflammation also exerts a significant role in the progression of DN. The progressive alternations in the glomerular function and structure result in the interstitial infiltration of inflammatory cells that exacerbate the DN progression through the secretion of pro-inflammatory and tissue remodeling cytokines. These molecules include tumor necrosis factor-alpha (TNF-α), interferon-γ (IFN-γ), IL-1, IL-6, and MCP-1, which can also induce oxidative stress via activating NADPH oxidase [8].

### Current and future biomarkers of DN

The early detection of DN is a critical medical demand, not only to predict and prevent DN progression but also to further enhance patients’ survival and decrease correlated morbidities. In clinical practice, DN diagnosis and prognosis are based on the presence of albuminuria, proteinuria, serum creatinine, blood urea nitrogen (BUN), and reduction of estimated glomerular filtration rate (eGFR), together with long-term diabetes. The DN severity is traditionally evaluated by measuring levels of urine albumin [urine albumin-to-creatinine ratio (UACR)]. The persistent microalbuminuria (30–300 mg/24 h) or macroalbuminuria (> 300 mg/24 h) has been widely used as a conventional biomarker of the early onset of DN and its progression to ESRD. However, in reality, kidney function is injured or even deteriorated before the detection of microalbuminuria/macroalbuminuria, and also there is conflicting evidence to the specificity and sensitivity of albuminuria [9, 10]. Moreover, the gold standard for the diagnosis of DN is mainly based on pathological alterations in renal biopsy, which is along with the drawbacks of an invasive approach and the inability to track DN progression [11]. Thus, exploring noninvasive and more specific and sensitive biomarkers of early stages of DN and development to the ESRD is necessary.

Over the last decade, considerable efforts have been made to find urine or serum biomarkers for noninvasive detecting early steps of DN and the progressive decline of renal function in diabetic patients. The search for biomarkers has often relied on serum or urinary biological molecules reflecting aspects of DN pathology. In the early stages of DN, a reduction in the number of podocytes often happens because of apoptosis or shedding of podocytes, which affects the glomerular filtration function. A growing body of research has shown the potentiality of urinary podocyte-specific proteins, including synaptopodin and nephrin, as noninvasive early biomarkers of glomerular damage in DN [12–20]. Of note, the urinary levels of these proteins were found to be extremely increased in DN patients even before proteinuria and indicated a strong correlation with UACR and eGFR [16–20].

Another pathological hallmark of DN complications is renal fibrosis resulting from ECM changes and mesangial expansion. Hyperglycemia upregulates the expression of TGF-β1 and TNF-α that are known to be the most important cytokines involved in tubulointerstitial fibrosis and glomerulosclerosis [21, 22]. These cytokines promote the cell apoptosis and accumulation of ECM in the mesangium, which reduces the rate of glomerular filtration and increases the permeability of tubules, leading to renal failure. As reported by independent studies, urinary and serum levels of both TGF-β1 [23] and TNF-α [24–26] are significantly higher in patients with microalbuminuria than in patients with normoalbuminuria and healthy subjects. Of note, urinary levels of these cytokines were found to be gradually elevated alongside the progression of DN, suggesting these cytokines as sensitive biomarkers in the early stage of DN [27–29].

Notably, tubulointerstitial damage plays a key role in the early stages of DN development, even before the evident glomerular dysfunction and proteinuria [15, 30]. The degree of tubulointerstitial damage is highly correlated with the renal prognosis. Thus, tubular markers of renal injury may be capable of reflecting the degree of sustained renal damage in diabetic patients. Neutrophil gelatinase-associated lipocalin (NGAL) is expressed in the renal tubular cells in response to kidney damage. NGAL has been documented as a promising new biomarker for the early stages of tubular renal injury [31–34]. Of note, the increased urinary NGAL correlates with the reduction in eGFR in diabetic patients with macro- or microalbuminuria [35]. Importantly, both urinary and serum NGAL indicate an elevating trend along with the decline in eGFR and albuminuria during the DN progression [36]. The urinary level of NGAL has also been found to be elevated even before the decline in traditional biomarkers (eGFR and albuminuria) in diabetic patients [3]. Thus, NGAL may be a useful biomarker for the early diagnosis of tubulointerstitial damage in patients with a high risk of progression to DN.

Despite the presence of the aforementioned potential biomarkers, but so far no biomarkers have been implemented in clinical care for the reason of lack of validation, and confirmation of their added value over that of the existing traditional biomarkers has yet to be proven [37]. Thus, alternative research is now focusing on emerging biomarkers to improve the sensitivity of biomarkers for predicting diabetic patients who will develop DN or are at a high risk of progressing to ESRD. These include circulating microRNAs (miRs) and extracellular vesicles (EVs) such as exosomes.

### Current and emerging therapies for DN

There is no cure available for DN and the current management relies on optimized blood pressure and glycemic control using agents with renoprotective properties. The renin–angiotensin–aldosterone system (RAAS) inhibitors (angiotensin-II receptor blockers [ARB] and angiotensin-converting enzyme [ACE] inhibitors) are the first-line blood pressure-lowering agents administrated for DN treatment [20–23]. The beneficial effects of RAAS inhibitors, such as captopril (ACE inhibitor) [38], irbesartan (ARB) [39, 40], and losartan (ARB) [41], have been documented in various randomized clinical trials. The RAAS blockade has been found to slow the rate of progression to albuminuria and decrease the risk of serum creatinine elevation and the complex outcome of ESRD or death [38–41]. The therapeutic efficacy of RAAS inhibitors in DN patients is because of their capability to decrease not only systemic blood pressure but also the glomerular hyperfiltration and the intraglomerular pressure by vasodilation of the efferent arteriole [42]. However, the administration of an ARB or ACE inhibitor cannot completely terminate the DN progression. Notably, a combination of ACE inhibitors and ARBs has also been found to be associated with an elevated risk of adverse effects such as acute kidney injury and hyperkalemia [43–45]. Recently, the novel mineralocorticoid receptor antagonists (MRAs), such as finerenone [46] and esaxerenone [47], have attracted much attention for intensifying the RAAS inhibition. An addition of MRAs to the standard ARB treatment or ACE inhibitor has been found to significantly reduce UACR, albuminuria, the decline in eGFR, and the risk of the complex outcome of renal failure or renal-caused death [46–48].

Moreover, glucagon-like peptide-1 (GLP-1) receptor agonists and sodium-glucose co-transporter 2 (SGLT2) inhibitors have shown anti-proteinuric/albuminuric and renoprotective effects beyond their glucose-lowering effects in DN patients, through the monotherapy or on the top of RAAS inhibition. Several randomized controlled trials have reported a significantly lower risk of incident or worsening nephropathy or death in T2DM patients who were treated with SGLT2 inhibitors empagliflozin [EMPA-REG OUTCOME trial] [49], canagliflozin [the CANVAS Program] [50], or dapagliflozin [the DECLARE-TIMI 58 trial [51] and DAPA-CKD trial [52]]. Recently, the CREDENCE trial indicated clear benefits of canagliflozin against elevated levels of the creatinine, the composite endpoint of ESRD, or renal-caused death in T2DM patients with macroalbuminuria [53]. Currently, the impact of empagliflozin on the progression of renal disease and the occurrence of death in diabetic and non-diabetic patients is being evaluated in the ongoing EMPA-KIDNEY trial (NCT03594110). Another type of glucose-lowering agents, GLP-1 receptor agonists have also shown an association with a lower risk of new-onset macroalbuminuria and a significant decrease in the progression of UACR in the macroalbuminuric condition, with moderate impacts on eGFR in T2DM patients [54–56]. Notably, an ongoing randomized controlled trial is currently being studied the long-term impacts of GLP-1 receptor agonist semaglutide on the progression to ESRD, rate of eGFR reduction, or death from renal disease (NCT03819153).

Nonetheless, many DN patients eventually progress to ESRD. Thus, there is an essential need for novel therapies that will refine renal function, decrease disease progression, and importantly enhance renal survival in DN. Currently, several therapeutic approaches are being investigated and may provide the foreseeable future therapies for DN. Generally, emerging treatments include the existing and under-investigation medications that target inflammatory, oxidant, or pro-fibrotic pathways.

Apoptosis signal-regulating kinase 1 (ASK1) is a key pathway associated with oxidative stress in DN. ASK1 can trigger the generation of pro-inflammatory and pro-fibrotic mediators leading to inflammation, matrix remodeling, fibrosis, and apoptosis [57]. Selonsertib (GS-4997) is an ASK1 inhibitor that has been found to improve kidney functional parameters such as eGFR, serum creatinine, and proteinuria in both animal models [58] and patients with renal disease (NCT02177786).

Moreover, phosphodiesterase inhibitors including pentoxifylline (NCT00663949) [59, 60], CTP-499 (NCT01487109, NCT01328821) [61], and PF-00489791 (NCT01200394) [62] are mainly recognized for their anti-inflammatory activity. These agents are associated with a significant decrease in the eGFR decline and UACR as well as a marked alleviation in levels of inflammatory markers including serum C-reactive protein (CRP) and urinary CCL2/MCP-1.

The pro-inflammatory chemokine (C–C motif) receptor type 2 (CCR2) and CCL2/MCP-1 play important roles in the pathogenesis of DN. The overexposure to the high glucose and/or filtered proteins induces the production and release of chemokines by tubular cells and podocytes, resulting in the recruitment of inflammatory cells and renal impairment [57]. The preliminary data from both animal models and clinical trials have demonstrated the renoprotective effects of chemokine inhibitors in DN. Of note, the CCR2 inhibitor CCX140-B [63] and CCL2 inhibitor NOX-E36 [64] have been found to improve proteinuria, the podocyte damage, the number of inflammatory macrophages, and the glomerular endothelial glycocalyx in the experimental mouse models of DN. On top of current standard treatments, CCX140-B (NCT01447147) [65] and NOX-E36 [66] were found to significantly slow the eGFR decline and reduce albuminuria in T2DM patients with nephropathy. Another potential target is the endocannabinoid system (ECS). Despite its major expression and function in the central nervous system, the ECS also plays a role in the kidney and especially in DN pathogenesis. The cannabinoid receptor 1 (CB1R) and 2 (CB2R) show opposite behaviors in DN. Podocytes overexpress CB1R in both human and investigational DN, whereas podocyte expression of CB2R is significantly downregulated in patients with advanced DN. Of note, CB1R signaling elevates the fibrogenesis, inflammation, and oxidative stress, while CB2R shows opposite impacts [67]. Both peripheral CB1R antagonists and CB2R agonists decreased albuminuria, inflammation, and renal fibrosis, and reversed alterations in the renal function in animals with persisted albuminuria [68, 69]. These agents are currently under clinical investigations to confer the proof of tolerability and safety, particularly CNS safety.

On the other hand, endothelins are the small vasoactive peptides that exert multiple pathophysiological effects, including damage to podocytes (nephron shedding, cytoskeletal disruption, and proteinuria), mesangium (proliferation and ECM accumulation), and tubulointerstitium (fibrosis) as well as inducing inflammatory cell infiltration [70]. In experimental studies, antagonists of endothelin receptors improved the renal morphology and function and reduced albuminuria through multiple mechanisms, including attenuated damage to mesangial cells, podocytes, renal tubules, and glycocalyx [71–74]. Of note, atrasentan is a selective endothelin-receptor antagonist with renoprotective features, which could reduce albuminuria in DN patients at a high risk of developing ESRD [75].

Besides approaches aimed to alleviate oxidative stress and inflammation in the kidney, novel potential therapeutic strategies based on stem cells, exosomes, and miRs are on the horizon with promising preclinical findings. With advances in stem cell technology, stem cell-based regenerative medicine has shown the potential as a therapeutic approach. There is a growing number of preclinical studies showing successful outcomes of stem cell transplantation for halting the progression of DN [76–92]. In brief, these studies provided evidence that stem cell therapy can improve functional indices, such as the elevation in glomerular filtration and the reduction in albuminuria and glomerulosclerosis. The studies demonstrated an improvement in renal histology and the suppressing of nephrocyte death, oxidative stress, inflammation, and renal fibrosis. Moreover, stem cell therapy was found to preserve renal mass, upregulate the expression of podocyte and tubular epithelial genes, augment levels of growth factors within the kidneys, reduce endothelium damage, and ameliorate tubular glucotoxicity by reducing the uptake of cellular glucose in the kidneys. These preclinical findings have been further supported by a clinical study (NCT02585622) that showed the stem cell transplantation could improve the eGFR, without significant treatment-related severe adverse events, in diabetic patients with progressive renal disease [93]. Recent studies suggest that the therapeutic potential of stem cells has been majorly dependent on their secretory capacity, particularly exosomes [94].

Exosomes are lipid bilayer EVs secreted by a broad range of cell types. These vesicles mirror the molecular content of donor cells and participate in cellular cross-talk between both neighboring and distant cells under physiological and pathological conditions. Exosome-mediated intercellular communication stems from its capacity in cell-to-cell transferring of biological information. miRs, a key functional cargo of exosomes, are principal negative regulators of the genome, which are mostly dysregulated in pathological conditions. The selective packaging and intercellular delivery of miRs by exosomes have encouraged deeper research of exosomal miRs (Exo-miRs), both as biomarkers and therapeutic agents [95–100]. A growing number of preclinical and clinical investigations indicates the impressive roles of Exo-miRs in DN progression. On the one hand, it has been recently shown that stem cell-derived exosomes contain renoprotective miRs posing therapeutic impacts on DN. On the other hand, various preclinical and clinical studies have reported that circulating Exo-miRs isolated from urine or bloodstream hold great potential as biomarkers in DN.

The present review highlights stem cell-derived Exo-miRs reported as the potential therapeutic tools for treating DN. In sum, an overview of the findings showed that exosomes derived from different sources of stem cells deliver distinct patterns of renoprotective miRs to the target cells that are injured in DN. The second part of this review article focuses on the studies that investigated the potentiality of circulating Exo-miRs as non-invasive biomarkers for the early diagnosis/prognosis of DN progression in diabetic patients.

## Methods

This review article aimed to seek renoprotective stem cell-derived Exo-miRs for treating DN as well as circulating Exo-miRs showing biomarker features for diagnosing/predicting DN progression in diabetic patients. To this end, a systematic literature search was performed in MEDLINE (http://www.ncbi.nlm.nih.gov/pubmed), Scopus (http://www.scopus.com), and Google Scholar (http://scholar.google.com), without any language restrictions, to identify all published articles dealing with the aims of the present study. Two independent searches were carried out from inception to July 2021 using the terms [(diabetic nephropathy OR diabetic renal disease OR diabetic kidney disease) AND (exosome) AND (microRNA OR miR) AND (stem cell)] as well as [(diabetic nephropathy OR diabetic renal disease OR diabetic kidney disease) AND (exosome) AND (microRNA OR miR) AND (circulation OR blood OR serum OR plasma OR urine)] in titles and abstracts. Based on the abstract, documents which include the following criteria were collected for the full-text screening: original articles reported the in vitro, in vivo, and clinical studies regarding the renoprotective stem cell-derived Exo-miRs in diabetic condition as well as serum/plasma or urinary-derived Exo-miRs showing biomarker capacity for detecting DN and its progression. The exclusion criteria were as follows: full text inaccessible; duplicate and non-original studies; not written in the English language; and only evaluated exosomes or miRs.

## Stem cell-derived Exo-miRs posing therapeutic effects on DN

**Fig. 1:**
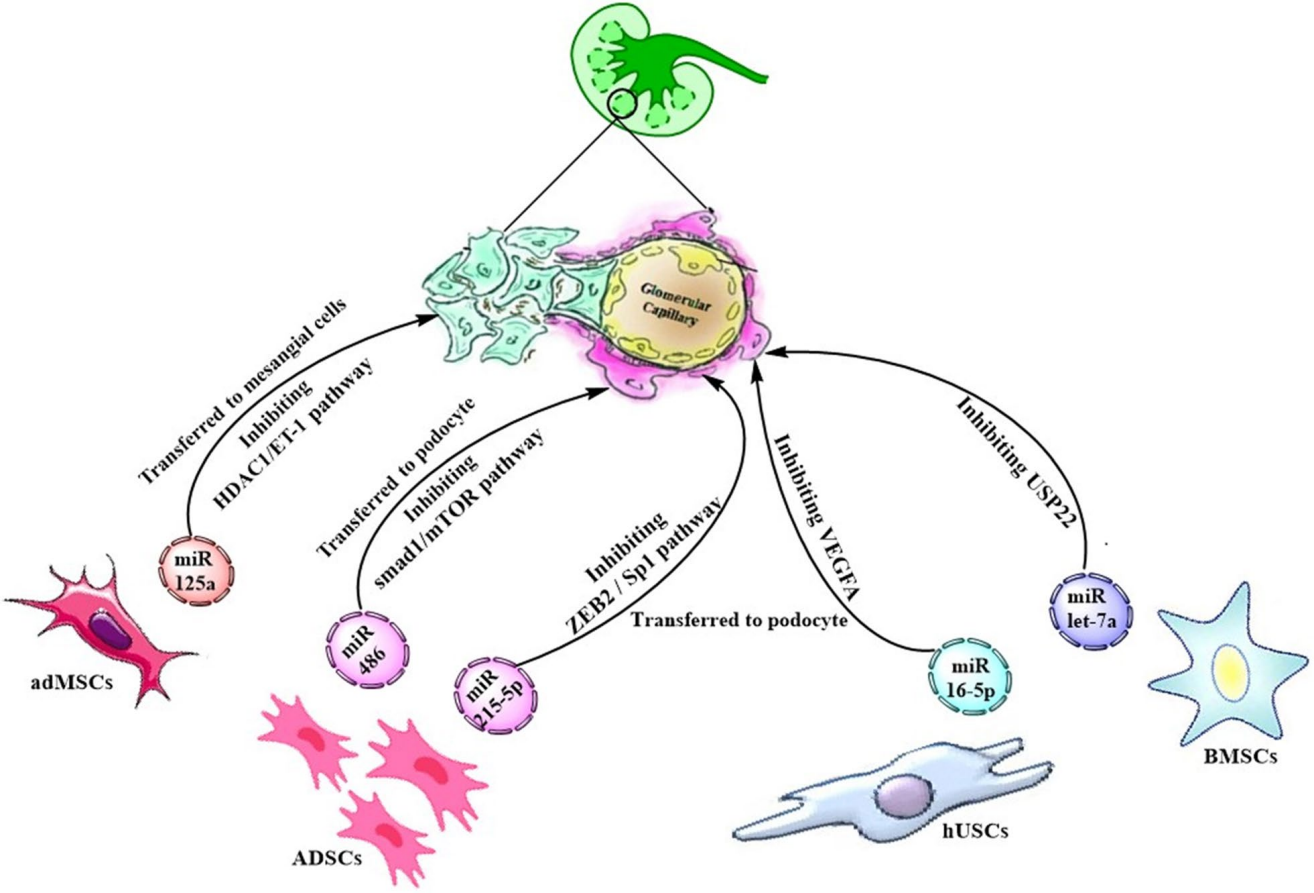
Stem cell-derived exosomes containing renoprotective miRs against diabetic nephropathy. The adMSCs-secreted exosomes transfer renoprotective miR-125a to injured mesangial cells where this miR directly binds to HDAC1 and further downregulates ET-1 expression, resulting in amelioration of mesangial hyperplasia. On the other hand, renoprotective exosomal microRNAs (Exo-miRs) secreted by ADSCs (Exo-miR-486 and Exo-miR-215-5p), hUSCs (Exo-miR-16-5p), and BMSCs (Exo-miR-let-7a) are transferred to injured podocytes, where these miRs suppress cell apoptosis and consequently inhibits renal damage. Adipose-derived mesenchymal stem cells, adMSCs; adipose-derived stem cells, ADSCs; human urine‐derived stem cells, hUSCs; microRNAs, miRs; bone marrow mesenchymal stem cells, BMSCs; histone deacetylase 1, HDAC1; endothelin-1, ET-1

Stem cell-derived exosomes have been found to not only recapitulate the therapeutic activities of parent cells but also provide advantages over them [101]. They are less complex and smaller than cells and have the potential to circumvent drawbacks of cell therapy, such as limited engraftment and poor survival and differentiation of stem cells caused by the diabetic microenvironment, risk of differentiation into unwanted cell lineages and formation of ectopic tissue, risk of tumorigenicity and genetic aberrations, as well as ethical and safety challenges [101]. Notably, exosomes have autonomous targeting capabilities and can home to a specific lesion tissue [101]. Exosomes have been found to internalize in a cell type-specific route that relies on recognition of exosomal surface ligands/receptors by the target cell or tissue. For instance, SDF-1α/CXCR4 interaction was exhibited to mediate the selective delivery of endothelial cell-derived exosomes to the kidney [102]. As discussed in the following subsections, exosomes released from various types of stem cells have been found to exert beneficial effects in DN by transferring renoprotective miRs to injured podocytes and mesangial and tubular cells (Table 1 and Fig. 1).

**Table 1:** Renoprotective Exo-miRs derived from stem cells

Source of stem cell	Exo-miR	In vitro target cell	In vivo clinical effects	Molecular target of Exo-miR	Refs
adMSC	miR-125a	Mesangial cells	Reducing the mesangial hyperplasia, the expansion rate of the mesangial matrix, and kidney fibrosis in DN rats	HDAC1	[107]
ADSC	miR-486	Podocyte	Improving the GFB function in DN mice	Smad1	[118]
	miR-26a-5p	Podocyte	ND	TLR4	[119]
	miR-215-5p	Podocyte	-	ZEB2	[123]
hUSCs	miR-16-5p	Podocyte	Improving the GFB function in DN rats	VEGFA	[130]
BMSCs	miR-let-7a	Podocyte	Improving the GFB function in DN rats	USP22	[136]
	miR-222	Mesangial cells	ND	STAT5	[106]
	miR-125b	Tubular cells	ND	TRAF6	[146]
	miR-let7c	Tubular cells	The reduction of the ECM accumulation and the amelioration of the fibrosis	TGF-β1	[147]

Not defined, ND; Adipose-derived mesenchymal stem cells, adMSCs; adipose-derived stem cells, ADSCs; human urine‐derived stem cells, hUSCs; bone marrow mesenchymal stem cells, BMSCs; exosomal microRNA, Exo-miR; Histone deacetylase 1, HDAC1; zinc finger E-box-binding homeobox-2, ZEB2; vascular endothelial growth factor A, VEGFA; signal transducer and activator of transcription 5, STAT5; TNF Receptor Associated Factor 6, TRAF6; toll-like receptor 4, TLR4; transforming growth factor beta 1, TGF-β1

### Stem cell-derived Exo-miRs posing protective effects on mesangial cells

The glomerular mesangial cells (GMCs) generate the mesangial matrix, provide the structural guard to the glomerular tuft, communicate with other glomerular cells via releasing soluble mediators, and assist the glomerular capillary flow by their contractile ability. Hyperglycemia promotes GMC activation, which commonly leads to immoderate cell proliferation and hypertrophy as well as the excessive production of the mesangial matrix, through the upregulating glucose transporters and an elevation in the entrance of glucose into the cells [103]. The mesangial matrix expansion leads to the glomerular capillary blockade and a progressive reduction in the filtration surface of the glomerulus [104]. If activation of GMCs is continuing, expansion of glomerular mesangium and the increased accumulation of the mesangial matrix in the interstitial space will cause progressive scarring and fibrosis of glomerular mesangium, a major hallmark of DN known as glomerulosclerosis [105]. TGFβ is the most relevant regulatory cytokine in the onset of renal fibrosis and collagen accumulation in GMCs under hyperglycemic conditions. Notably, it has been shown that EVs secreted from bone marrow mesenchymal stem cells (BMSCs) protect GMCs from the high glucose (HG)-induced damage via the transfer of miR-222. By targeting and downregulating STAT5, miR-222 was found to significantly reduce TGF-β expression and collagen production within GMCs [106]. In addition, it was recently reported that exosomes released from adipose-derived mesenchymal stem cells (adMSCs) could significantly inhibit the excessive proliferation of HG-treated GMCs mimicking a DN-like condition in vitro. Of note, IL-6, a typical autocrine growth factor inducing glomerular damage and mesangial hyperplasia, was found to be highly increased in HG-treated GMCs. The adMSC-exosome treatment could significantly inhibit the HG-induced IL-6 expression in GMCs [107]. It was further supported by the in vivo study that showed the treatment with adMSC-exosomes effectively alleviated mesangial hyperplasia, the expansion rate of the mesangial matrix, and kidney fibrosis in STZ-induced DN rats. These effects were along with a significant reduction in serum creatinine, urinary protein, and UACR, as well as a considerable amelioration of kidney pathological symptoms including capillary lumen shrinking, infiltration of inflammatory cells, and renal tubular damage [107]. Notably, elevated levels of collagen I and fibronectin contribute to the renal fibrosis and the impaired renal function and are key biomarkers for the mesangial matrix expansion [108]. Of note, adMSC-exosomes were found to significantly inhibit the upregulation of these fibrosis-related factors in the kidney of DN rats and in vitro in HG-treated GMCs [107], supporting the protective effect of adMSC-exosomes on renal fibrosis and function. Importantly, miR-125a was found to be responsible for the renoprotective effects mediated by adMSC-exosomes. Notably, the downregulation of miR-125a inhibited the protective effects of adMSC-exosomes in the DN rat model and in HG-treated GMCs [107]. miR-125a had also been already shown by other studies to be carried by MSC-exosomes [109] and to play a key role in DN patients, with a significant preventive effect on the disease progression [110]. The histone deacetylase 1 (HDAC1) has been found to be a direct mRNA target of miR-125a in the kidney tissues in rats and in GMCs [107]. HDAC1 can exacerbate DN progression through upregulating endothelin-1 (ET-1). adMSC‒Exo-miR-125a was found to protect the kidney injury in DN rats through inhibiting the HDAC1/ET-1 axis [107]. Supporting is convincing evidence that shows a firm association between ET-1 with diabetes and its complications. ET1 has been reported to promote insulin resistance [111], to elevate the glomerular permeability and in turn elevate the serum level of creatinine [112], to correlate with the proteinuria level in DN patients [113], to induce proliferation of mesangial cells and accumulation of mesangial matrix [114], and to play a pro-fibrotic role in diabetic complications [115]. In conclusion, abovementioned findings indicate that exosomes released from adMSC provide the promising tool to ameliorate the renal fibrosis and improve the kidney function through transferring miR-125a to GMCs where directly binds to HDAC1 and further downregulates ET-1 expression.

### Stem cell-derived Exo-miRs posing protective effects on podocytes

The glomerular filtration barrier (GFB) is specialized to permit substantial filtration of water and solutes in the kidney. The GFB dysfunction is a typical clinical symptom of DN, which is accompanied with microalbuminuria in the early stage of DN, proteinuria progression, and kidney dysfunction over numerous years to decades, resulting in ESRD [116]. Podocytes include a class of uniquely differentiated visceral epithelial cells covering the outside of the GBM, which act as the final protective barrier of the kidney and play an essential role in maintaining the function of GFB. An important event in the development of DN includes the HG-mediated apoptosis of glomerular podocytes, which can result in GFB dysfunction and proteinuria [117]. As discussed in the following subsections, stem cell-derived Exo-miRs released from adipose-derived stem cells (ADSCs), human urine‐derived stem cells (hUSCs), and BMSCs can effectively inhibit the podocyte injury and thereby ameliorate the GFB dysfunction in DN.

#### ADSC‒Exo-miRs

There are reports that indicate ADSCs via secreting Exo-miRs, which exert protective effects against the podocyte injury, can improve the glomerular filtration in DN. A recent in vivo study reported that the administration of ADSC-derived exosomes could significantly improve the GFB function through the protective effect against the podocyte apoptosis in DN mice. This effect was along with a reduction of the serum creatinine, urine protein, and BUN, as well as the relieved pathological changes of kidney tissues, including the excessive proliferation of GMCs, accumulation of mesangial matrix, and GBM thickness [118]. [It should be noted that the amelioration of mentioned renal pathological changes is not surprising because podocytes can communicate with other glomerular cells, in which podocyte damage may promote proliferation of GMCs [105]]. The in vivo findings were further supported by the in vitro study that showed ADSC-exosomes could effectively suppress the HG-induced apoptosis in mouse podocyte MPC5 [118]. Another study reported that exosomes released by ADSCs contain a high level of miR-26a-5p that is transferred to glomerular podocytes and efficiently ameliorates the pathological symptoms of DN in diabetic mice [119]. The in vitro study indicated that ADSC-derived Exo-miR-26a-5p could protect HG-induced podocytes from apoptosis and improve their viability by targeting TLR4, downregulating VEGFA, and silencing the NF-κB pathway [119]. Notably, the thickening of the GBM and the expansion of the mesangial matrix are associated with podocyte apoptosis and autophagic flux suppression. The impaired autophagic function is an indicator of podocyte apoptosis in DN models in vitro and in vivo [120]. The mechanistic target of rapamycin (mTOR) signaling, a key regulator of autophagy, is hyperactivated in DN and plays a pivotal role in the process of podocyte apoptosis and the decreased rate of glomerular filtration [121]. Of note, ADSC-exosomes were found to improve autophagy flux and decline podocyte apoptosis by suppressing the activation of mTOR signaling in HG-induced MPC5 cells and DN mice [118]. Further study revealed that miR-486 is a key mediator in the process of ADSC-Exo-mediated alleviation of DN symptoms in vitro and in vivo [118]. Mechanistically, ADSC-Exo-miR-486 was found to directly target and downregulate the expression of Smad1, thereby suppressing the activation of the mTOR pathway, resulting in the promotion of autophagy flux and the inhibition of podocyte apoptosis induced by HG [118].

Furthermore, the HG-mediated podocyte damage can also be characterized by the epithelial-mesenchymal transition (EMT) and the migration resulting in the process of podocyte loss. This process is known as the important causative factor of GFB destruction and proteinuria production, leading to DN development [122]. Notably, ADSCs-exosomes were found to inhibit the HG-induced EMT progression and migration of podocytes through the shuttling miR-215-5p to podocytes. In mechanism, miR-215-5p was shown to mediate such an effect through suppressing the expression of zinc finger E-box-binding homeobox-2 (ZEB2) [123]. This can be supported by other studies that showed miR-215 could suppress cancer cell migration and apoptosis by directly inhibiting ZEB2 expression (124). Mechanistically, ZEB2 can interact with the transcription factor Sp1 to activate the expression of mesenchymal genes, resulting in the promotion of the EMT process and cell migration [125]. ZEB2 can also directly bind to conserved E2 boxes of the E-cadherin promoter to repress E-cadherin expression, thereby accelerating the EMT [126].

In conclusion, ADSCs-exosomes can transport miR-26a-5p, miR-486, and miR-215-5p to podocytes where these miRs protect against cell apoptosis and migration and the EMT process, thereby improving glomerular filtration in DN condition. Potentially, such ADSCs-derived Exo-miRs can serve as promising therapeutic candidates for DN treatment in the future.

#### hUSC‒Exo-miRs

hUSCs have shown significant potential in the treatment of diabetic diseases [127, 128]. The protective role of hUSCs‐derived exosomes against kidney injury in DN has been initially reported by the study that revealed the intravenous injection of hUSC‐exosomes in STZ‐induced rats could reduce urine volume and urinary microalbumin excretion as well as suppress podocyte apoptosis by inhibiting overexpression of caspase-3 [129]. Further studies indicated that Exo-miR-16-5p secreted by hUSCs could effectively alleviate podocyte apoptosis and enhance podocyte proliferation in the DN rats and in vitro under HG condition, mechanistically through inhibiting the expression of VEGFA [130]. VEGFA, a growth factor known for its role in angiogenesis as well as cell permeability and survival, is abnormally expressed in kidney tissues to a wide range of renal diseases [131]. The increased glomerular level of VEGFA in DN mice was found to be attributed to the impact of HG on the VEGFA expression in podocytes [132]. The upregulation of VEGFA in podocytes could cause podocyte apoptosis, abnormality in glomerular selectivity and filtration, and a reduction in renal function in cases of DN [133]. These findings can further support VEGFA-mediated protective effects of hUSCs‒Exo-miR-16-5p against DN-induced podocyte injury [130], presenting a new window for future research regarding DN treatment.

#### BMSC‒Exo-miRs

BMSCs play an important role in the replacement therapy of DN [77], and impaired BMSCs derived from diabetic animals have been found to exert no therapeutic impact on DN [134]. A preclinical study in a mouse model of DN indicated that the treatment with BMSC-derived EVs remarkably improved functional parameters, such as the plasma creatinine, BUN, and albumin/creatinine excretion [135]. Importantly, renal fibrosis was significantly inhibited and reverted in EV-treated mice [135]. An association was identified between the anti-fibrotic impact of BMSC-EVs and the downregulation of various pro-fibrotic genes in kidney tissues [135]. A comparative analysis of the miR content of BMSC-EVs highlighted some specific patterns of miRs that target predicted pro-fibrotic genes [135]. There is evidence that shows Exo-miR-let-7a implicates the protective role of BMSCs in DN [136]. miR-let-7a targets the ubiquitin-specific peptidase 22 (USP22) that participates in the pathological development of DN via modulating the expression of TGF-β [137]. Notably, TGF-β induces podocyte apoptosis during glomerulosclerosis [138] and is an important player in renal fibrosis [139]. Of note, the downregulation of miR-let-7a and the overexpression of USP22 have been detected in DN patients [140, 141], renal tissues of DN rats [136], as well as podocytes and mesangial cells under the HG condition [142, 143]. It was shown that the injection of BMSCs-Exo-miR-let-7a could significantly increase levels of miR-let-7a in renal tissues and thereby improve renal function parameters in DN rats [136]. Indeed, increased Exo-miR-let-7a, through directly targeting and repressing overexpressed USP22 in glomerular cells such as podocytes, could inhibit renal cell apoptosis, which was accompanied with a reduction in serum creatinine, BUN, and blood lipid indices in DN rats [136]. To sum up, BMSCs-exosomes deliver miR-let-7a to renal cells and, whereby, inhibit the cell apoptosis through downregulating USP22, thereby exerting a protective role in DN. This emphasizes potentiality of BMSC-derived Exo-miR-let-7a as the therapeutic tool. However, more studies should be done for taking insights into the underlying molecular mechanisms to develop such BMSC-derived Exo-miR as a novel therapeutic tool for DN treatment.

### BMSC‒Exo-miRs posing protective effects on renal tubular epithelial cells

The damage to tubular epithelial cells contributes to interstitial fibrosis in kidney disorders, such as DN. In diabetic animals, treatment with BMSC-derived exosomes has been found to prevent apoptosis and damage of tubular epithelial cells, abolish interstitial fibrosis, and improve kidney function [144, 145] by activating autophagy associated with inhibition of the mTOR signaling pathway [145]. Of note, it was shown that injection of BMSC-derived exosomes in the kidney could improve the histopathological feature of DN in the form of reduced atrophic alterations, vacuolation, degeneration, and inflammatory cell infiltration of proximal tubule epithelial cells, together with the renal fibrosis [144]. There is a growing body of strong evidence that shows the protective effect of BMSC-derived exosomes on tubular epithelial cells is attributed to their miR content. Notably, it was reported that BMSC-Exo-miR-125b could significantly suppress apoptosis and promote autophagy in HG-treated human embryonic kidney epithelial cells via targeting the TRAF6/Akt axis [146]. Another in vitro study indicated that BMSC-Exo-miR-let7c could be selectively transferred to damaged kidney tubular epithelial cells and suppress the upregulated expression of the ECM molecules, including types 1α1 and IVα1 collagen and α-smooth muscle actin (α-SMA), through inhibiting TGF-β1 signaling pathway [147]. These effects were found to be companied with the reduction of the ECM accumulation and the amelioration of the fibrosis in a mouse model of renal fibrosis treated with BMSC-Exo-miR-let7c [147]. To sum up, exosomes released from BMSCs can protect renal tubular epithelial cells and thus prevent interstitial fibrosis through delivery of miR-125b and miR-let7c.

## Circulating Exo-miRs posing potential biomarkers in DN

### The rationale for Exo-miR-based biomarkers

Due to the relatively fast and easy isolation and measurement as well as the high specificity and sensitivity, circulating exosome-based biomarkers such as Exo-miRs have growingly received great attention for their non-invasive diagnostic potentials in various diseases such as DN. Exo-miRs can be detected and isolated from various bodily fluids, particularly urine [148] and serum/plasma [149]. Circulating Exo-miRs possess great characteristics as an ideal source for experimental and clinical biomarkers compared to free-miRs [150]. The biological function is a primary requirement for a candidate biomarker; Exo-miRs are actively secreted and delivered to the distant recipient cells and form an intercellular communication, thereby modulating the function of recipient cells [151]. In the case of specificity, Exo-miRs are selectively packaged and mirror the cellular origin and its (patho)physiological states, thereby enabling recognition of their source and discriminating normal and diseased cells [152], whereas it is a challenge to determine the cell source of a specific circulating free-miR [153]. Moreover, miRs are highly enriched in serum- and urine-derived exosomes, thereby providing higher sensitivity than free-miRs [154]. At variance with free-miRs, Exo-miRs are also in a considerable stable form since the lipid membrane of exosomes protects them against RNase degradation in biological fluids and the storage environment. Exo-miRs are stable during long-term storage and show resistance to multiple freeze–thaw cycles. Notably, urinary Exo-miRs are stable at 4 °C up to 24 h for shipping before being stored at − 80 °C and can be stable for one year in urine once stored at − 80 °C [155].

### Urinary Exo-miRs as the potential biomarker in DN

**Fig. 2:**
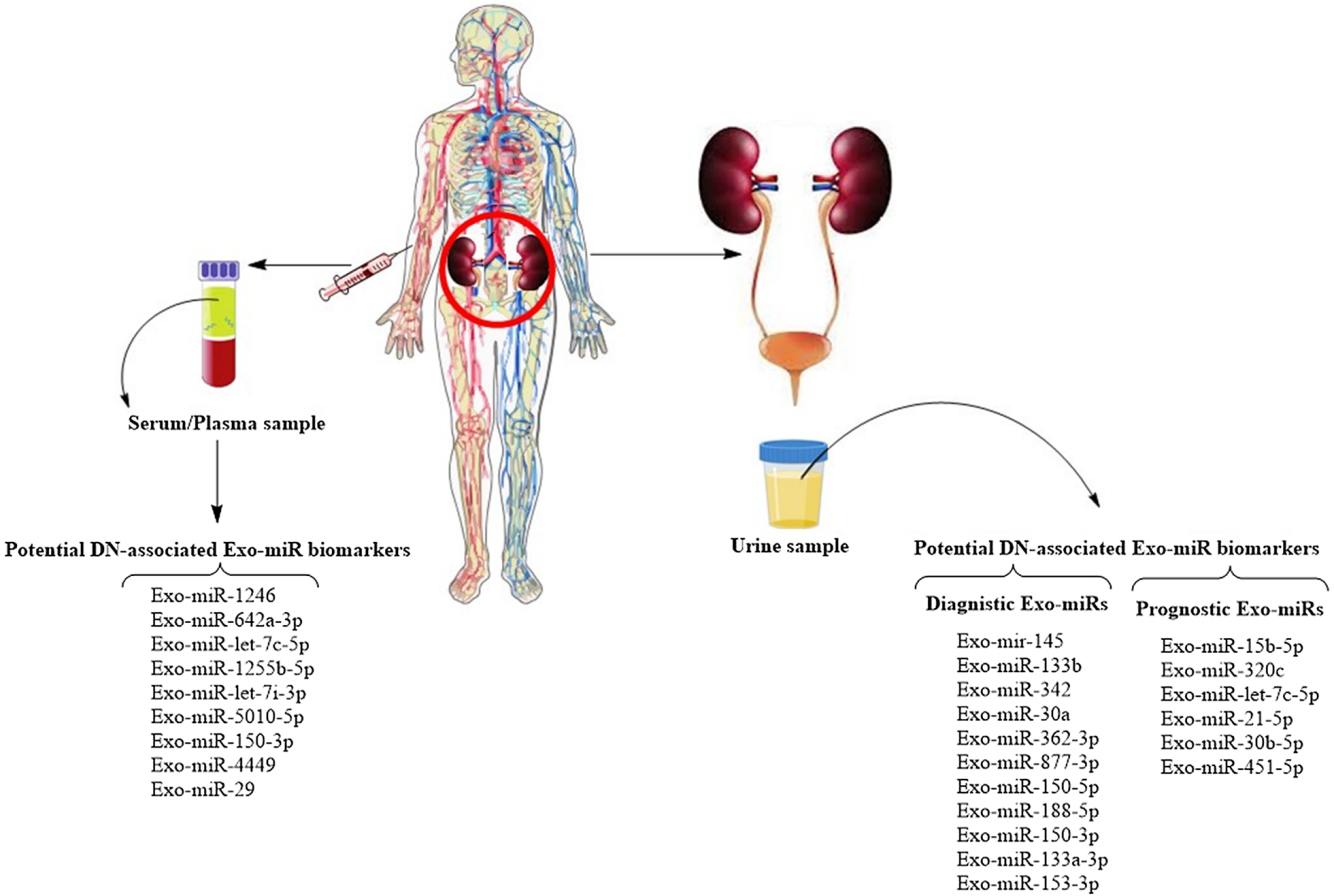
Potential DN-associated Exo-miRs biomarkers isolated from the plasma/serum and urinary exosomes

Urinary exosomes contain a wide array of miRs differentially expressed in DN (Table 2 and Fig. 2). Although profiling of total urine miRs is easier to carry out and less time-consuming with resultant superiority for translation in clinical use, the analysis of urinary Exo-miRs is more accurate since a substantial part of total urinary miRs can originate from other sources, like damaged cells of the urinary tract or plasma miRs passing the GFB, rather than from the kidney tissue [156]. This decreases the relevance of analyzing total urinary miRs for discovering biomarkers in kidney pathophysiology. As illustrated in the following subsections, there is convincing evidence that suggests the distinctive signature of urinary Exo-miRs might have the potential as early diagnostic and prognostic biomarkers for DN progression in T1DM or T2DM patients.

**Table 2:** Potential circulating Exo-miR biomarkers of DN

miRs (exosomal level)	Species	Type of DN	Cell source	Molecular target	Refs
**Urinary exosomal miRs**					
miR-145 (+)	Human, mouse, cell	Type 1 DN	Mesangial cell	ND	[156]
miR-15b (+) miR-34a (+) miR-636 (+)	Human	Type 2 DN	ND	ND	[164]
miR-4534	Human	Type 2 DN	podocyte	FOXO1	[165]
miR-320c (+)	Human	Type 2 DN	ND	TSP-1	[167]
miR-let-7c-5p (+)	Human	Type 2 DN	ND	TGF-β	[172]
miR-362-3p (+) miR-877-3p (+) miR-150-5p (+) miR-15a-5p (‒)	Human	Type 2 DN	ND	AMPK, p53, mTOR	[163]
miR-21-5p (+) miR-30b-5p (‒)	Human	Type 2 DN	ND	ND	[175]
miR-15b-5p (+)	Human, mouse, cell	Type 2 DN	Mesangial cell	BCL-2	[166]
miR-188-5p (+) miR-150-3p (+) miR-133a-3p (‒) miR-153-3p (‒)	Human	Nephrotic-range proteinuria	ND		[111]
miR-133b (+) miR-342 (+) miR-30a (+)	Human	ND	ND	TGF-β1	[158]
miR-451-5p (+)	Rat	ND	ND		[186]
miR-483-5p (+)	Mouse	Type 1 and type 2 DN	Tubular cell	MAPK and TIMP2	[187]
**Serum exosomal miRs**					
miR-4449 (+) miR- 1246 (+) miR-642a-3p (+) let-7c-5p (+) miR-1255b-5p (+) let-7i-3p (+) miR-5010-5p (+) miR-150-3p (+)	Human	ND	ND	MAPK pathway TGF-β pathway Integrin-VEGF pathway Olfactory Pathway AP-1and NF-κB network	[176] [178]
Exo-miR-29 (+)	Human	Type 1 DN	ND	Serine/threonine-protein kinase WINK3 gene	(181)

(+) and (‒) show upregulation and downregulation, respectively

Not defined, ND; TSP-1, Thrombospondin 1; VEGF, vascular endothelial growth factor; AP-1, activator protein-1; NF-κB, nuclear factor-κB; MAPK, mitogen-activated protein kinase; TGF-β, transforming growth factor beta; DN, diabetic nephropathy; the tissue inhibitor of metalloproteinases 2, TIMP2

#### Potential urinary Exo-miR biomarkers for the early diagnosis of DN

In a study on T1DM patients, the miR profiling of urinary exosomes indicated a differential expression of urinary Exo-miRs in two firmly matched groups of patients with normo- and microalbuminuria [156]. Of note, patients had a low degree of microalbuminuria and normal renal function, indicating that abnormalities in the expression of urinary Exo-miRs appeared in an early stage of DN [156]. Accordingly, validation of profiling data confirmed that the urinary level of Exo-miR-145 was markedly increased in microalbuminuric patients as compared to normoalbuminuric T1DM patients and healthy controls [156]. This result was further supported with a subsequent in vivo study that indicated the miR-145 level was significantly elevated in both urinary exosomes and the glomeruli isolated from mice with early experimental DN [156]. miR-145 is known as a glomerular marker of mesangial cells [157]. The in vitro study [156] revealed that the HG treatment can promote the secretion of miR-145-enriched exosomes by mesangial cells, showing the mesangial cell origin of urinary exosomes carrying an increased level of miR-145 in DN patients. Thus, the elevated level of urinary Exo-mir-145 shows a pathophysiological relevance in the progression of DN and can provide a candidate biomarker for the early diagnosis of type 1 DN.

Besides, there have also been studies that reported potential urinary Exo-miRs for diagnosing early signs of onset or the adverse progression of type 2 DN. In a cohort study on T2DM patients [158], urinary Exo-miR-133b, Exo-miR-342, and Exo-miR-30a were detected to be highly overexpressed not only in macro- and microalbuminuric patients but also in normoalbuminuric subjects before albuminuria. This suggests the practicality of such urinary Exo-miRs as early molecular indicators prior to the initiation of albuminuria. These urinary Exo-miRs showed a significant positive correlation with renal failure parameters including serum creatinine, eGFR, and UACR. Moreover, Insilco data analysis via “pathway enrichment analysis” identified them as negative regulators of TGF-β1 [158]. Supporting is other studies showing involvement of these miRs in diabetic or renal diseases through targeting TGF-β1 signaling pathways [138, 159–162]. Taken together, the aforementioned findings indicate that the urinary Exo-miR-133b, Exo-miR-342, and Exo-miR-30a represent potential biomarkers for early diagnosis and risk stratification of type 2 DN. Potential urinary Exo-miRs for early diagnosis of type 2 DN were also reported by another study that showed the increased expression of three urinary Exo-miRs (miR-362-3p, miR-877-3p, and miR-150-5p) and the reduced expression of one (miR-15a-5p) in incipient T2DM patients with macroalbuminuria compared to those without macroalbuminuria [163]. Of note, these miRs were predicted to regulate pathways involved in DN processes, including AMP-activated protein kinase (AMPK), mTOR, and the p53 (apoptosis-induced nuclear transcription factor) pathways [163]. Another study on T2DM patients revealed the significant increase in urinary levels of a panel of Exo-miRs including Exo-miR-15b, Exo-miR-34a, and Exo-miR-636 in both DN and normoalbuminuric patients [164]. Notably, dysregulation of this panel of urinary Exo-miRs indicated a significant correlation with age, body mass index (BMI), HBA1c, hypertension, serum creatinine, and UACR [164]. These Exo-miRs were found to contribute to the DN pathogenesis through targeting critical pathways affected in DN, such as glucose metabolism, cell proliferation and apoptosis, cytokine release and growth factor signaling, as well as nephrogenesis and renal fibrosis [164]. Importantly, the diagnostic value of the urinary Exo-miR-based panel was strongly higher than that of the individual miR, reaching 100% sensitivity in diagnosing DN [164]. Thus, this urinary Exo-miR panel was suggested as a potential biomarker with high sensitivity and specificity in the early diagnosis of DN [164]. In the other study, profiling of urinary Exo-miRs indicated that miR-4534 was highly upregulated in DN patients compared to T2DM patients and healthy volunteers [165]. Importantly, the urinary level of Exo-miR-4534 showed a firm correlation with microalbuminuria in DN patients, while there was no correlation in the T2DM patients [165]. This suggests a possible role of Exo-miR-4534 in the early formation of microalbuminuria. The functional analysis indicated that the FOXO1 signaling pathway is a target of miR-4534 in DN. miR-4534 was found to involve in the DN progression by activating the FOXO/BNIP3/Atg12-mediated inflammatory pathway and worsening the podocyte damage [165]. FOXO1 has a role in the regulation of autophagy. It suppresses the expression of Atg14 to destroy the autophagy-lysosomal fusion, thereby inducing autophagy and apoptosis leading to vascular complications in diabetes [165].

Significantly, nephrotic-range proteinuria presents the most deleterious figure of proteinuria in diabetic patients. The analysis of urinary Exo-miR profile in nephrotic, renal biopsy-proven DN patients revealed the highest upregulation with miR-188-5p and miR-150-3p as well as the highest downregulation with miR-133a-3p and miR-153-3p. The functional analysis and the target-gene prediction of these miRs verified that they contribute to novel and known pathways of DN, supporting their pathologic role and potential as the biomarker in DN patients with nephrotic-range proteinuria [111].

#### Potential urinary Exo-miR biomarkers for the prognosis/prediction of DN

A recent study [166] indicated that both diabetic mice and T2DM patients with microalbuminuria had higher urinary levels of Exo-miR-15b-5p when compared with normoalbuminuric patients and healthy subjects. Notably, a mean follow-up period of 2.4 years indicated that urinary levels of Exo-miR-15b-5p are negatively correlated with eGFR and positively correlated with UACR and a rapid decline in renal function in T2DM patients. The in vitro study revealed that miR-15b-5p participates in the HG-mediated kidney injury by inducing mesangial cell apoptosis via targeting BCL-2 [166]. Moreover, in two independent cohort studies on T2DM patients [167], the expression profiling of urinary Exo-miRs indicated a differential signature of miRs in patients with microalbuminuria (stages 3 and 4 DN) compared to normoalbuminuric patients. Notably, the miR profile analysis showed a robust upregulation of miR-320c in urinary exosomes of patients with microalbuminuria [167]. Importantly, urinary levels of Exo-miR-320c were found to have negative and positive graded correlations with eGFR and UACR, respectively [167]. The increased level of urinary Exo-miR-320c might be a compensatory response to the over-activated TGF-β signaling pathway during DN progression. Indeed, miR-320c downregulates the TGF-β signaling via targeting thrombospondin 1 (TSP-1) [167] that is a key activator of TGF-β in renal fibrosis [168, 169] and shows an elevated expression in the glomeruli of DN patients [170, 171]. Another study on T2DM patients [172] showed that the level of miR-let-7c-5p was significantly elevated in urinary exosomes of DN subjects compared with patients without nephropathy as well as healthy controls. The urinary levels of Exo-miR-let-7c-5p also indicated a significant correlation with the eGFR and DN progression [172]. It can be further supported by other studies that reported the miR-let-7 family members (let-7b and let-7c) play functional roles in renal fibrosis [173, 174] and attenuate the renal fibrosis in DM through downregulating the TGF-β pathway [147]. Thus, it is likely that the increased level of urinary Exo-let-7c-5p is due to a physiological response to limit the renal fibrosis in DN. Another study also reported a high upregulation of miR-21-5p and a high downregulation of miR-30b-5p in urinary exosomes isolated from T2DM patients with DN and the poor renal function, suggesting them as promising predictors of the progression of early-stage DN to the renal failure [175]. In sum, these findings suggest urinary Exo-miR-15b-5p, Exo-miR-320c, Exo-let-7c-5p, Exo-miR-21-5p, and Exo-miR-30b-5p as the potential biomarkers for prognosing the severity and the adverse progression of the renal injury in type 2 DN.

### Serum Exo-miRs as potential biomarkers in DN

In addition to the urine Exo-miRs analysis, reports regarding the analysis of serum Exo-miRs exist as well. Evaluating the profile of serum Exo-miRs in healthy volunteers and diabetic patients with and without nephropathy indicated that, among differentially expressed Exo-miRs, eight miRs (miR-1246, miR-642a-3p, let-7c-5p, miR-1255b-5p, let-7i-3p, miR-5010-5p, miR-150-3p, and miR-4449) were DN-specific and their levels were significantly and uniquely increased in circulating exosomes [176]. Importantly, serum levels of these Exo-miRs were strongly correlated with the degree of albuminuria [176]. Notably, according to pathway analysis using miRSystem and DIANA-miRPath [176], these Exo-miRs were predicted to involve in the pathways and molecular targets previously reported to play a distinct pathogenic role in the progression of DN. These include the mitogen-activated protein kinase (MAPK) signaling pathway, the integrin-VEGF axis [177], the olfactory signaling pathway, and the activator protein-1 (AP-1) and nuclear factor-κB (NF-κB) transcription factor network [176]. Of note, among these miRs, miR-4449 was highly overexpressed in DN patients compared to patients without DN [176]. It is further supported by a recent study that showed miR-4449 is enriched in the serum exosomes of DN patients [178]. The functional analysis indicated that the overexpression of miR-4449 promotes inflammation, oxidative stress, and pyroptosis [178]. The other studies have also shown a significant correlation between the miR-4449 and the degree of albuminuria and suggested its potential as a novel biomarker for DN [179, 180]. These findings suggest serum Exo-miR-4449 as the promising biomarker candidate for the diagnosis/prognosis of DN.

Further, a cross-sectional case–control study including children and adolescents with T1DM [181] indicated that serum levels of Exo-miR-29 were significantly increased in subjects with microalbuminuria compared to subjects without microalbuminuria and healthy controls [181]. Exo-miR-29 was predicted to target the serine/threonine-protein kinase WINK3 gene that participates in glucose transporter mechanisms in the insulin signaling pathway as well as the beta-cell signaling and survival [181]. It was further supported by “enriched pathway analysis” that showed Exo-miR-29 targets numerous molecular and signaling pathways included in the insulin signaling pathway as well as the renal and nephron epithelium development [181]. This is in agreement with other studies that reported the regulatory role of miR-29 in the glucose homeostasis and the insulin action [182] and also showed increased serum levels of this miR in T1DM children and adult T2DM patients [183–185], which was associated with pathogenesis and progression of DN [183]. Notably, the regression analysis after adjustment of age, sex, BMI, disease duration, and lipid profile showed a strong association between the serum level of Exo-miR-29 with T1DM and persistent microalbuminuria [181]. Among patients with persistent microalbuminuria, the level of UACR was significantly higher in subjects with miR-29 overexpression than in those who did not exhibit overexpression [181]. Evaluating the differentiation between T1DM patients with and without persistent microalbuminuria confirmed that circulating Exo-miR-29 might represent a potential blood-based biomarker for early diagnosis of the DN in pediatric T1DM patients [181].

### Novel Exo-miR biomarkers based on the animal studies

An in vivo study on STZ-induced diabetic rats indicated that the level of urinary Exo-miR-451-5p was highly increased, by > 1000-fold, early on during the course of diabetes, before the renal damage. Notably, the increase in urinary levels of Exo-miR-451-5p was found to be a more sensitive predictor of DN when compared to albumin excretion [186]. This can support the potential usefulness of urinary Exo-miR-451-5p as a sensitive prognostic biomarker, instead of albumin excretion levels, to serially monitor the early renal damage in diabetic patients. However, further investigations are warranted to address this claim in human subjects.

Another in vivo study showed that the miR-483-5p expression was reduced in kidney tissues of type 1 and type 2 diabetic mice and HG-stimulated tubular epithelial cells, while it was increased in the urinary exosomes [187]. miR-483-5p was found to inhibit expressions of fibrosis-related genes in vitro and alleviate the renal interstitial fibrosis in vivo, through targeting and suppressing MAPK and the tissue inhibitor of metalloproteinases 2 (TIMP2) in renal tubular epithelial cells under HG conditions [187]. Importantly, HNRNPA1-mediated exosomal sorting transported cellular miR-483-5p out of tubular epithelial cells into the urine, thus reducing the inhibitory impact of cellular miR-483-5p on MAPK1 and TIMP2 mRNAs, and ultimately boosting the ECM accumulation and the progression of DN-induced renal interstitial fibrosis [187]. Thus, Exo-miR-483-5p has a valuable potential to be further assessed as a biomarker for the early diagnosis of renal fibrosis in DN.

## Conclusions

Despite numerous years of attempts, effective therapeutic approaches and fruitful diagnosis/prognosis biomarkers for DN remain still elusive. Endocrinologists and nephrologists are constantly searching for new therapeutic tools as well as for novel strategies to enhance their knowledge for rapid and accurate diagnosing of kidney disorders resulting from diabetes.

The present review of preclinical and clinical studies can conclude that stem cell-derived Exo-miRs provide promising therapeutic tools, while the differential expression of circulating Exo-miRs represents emerging biomarkers for DN. Stem cell-derived exosomes have shown beneficial effects in DN by transferring renoprotective miRs to injured renal cells. BMSC-secreted exosomes have been found to deliver renoprotective miRs to injured renal cells including podocytes (Exo-miR-let-7a), mesangial cells (Exo-miR-222), and tubular cells (Exo-miR-125b and Exo-miR-let7c). Moreover, exosomes secreted by adMSCs could deliver renoprotective miR-125a to injured mesangial cells, while renoprotective Exo-miRs secreted by ADSCs (miR-26a-5p, miR-486, and miR-215-5p), and hUSCs (Exo-miR-16-5p) are transferred to injured podocytes. Besides, urinary Exo-mir-145 in T1DM, urinary Exo-miR-133b, Exo-miR-342, Exo-miR-30a, Exo-miR-15b, Exo-miR-34a, and Exo-miR-636, and Exo-miR-4534 in T2DM, and urinary Exo-miR-188-5p, Exo-miR-150-3p, Exo-miR-133a-3p, and Exo-miR-153-3p in diabetic patients with nephrotic-range proteinuria provide candidate promising biomarkers for the early diagnosis of DN. In addition, urinary Exo-miR-15b-5p, Exo-miR-320c, Exo-let-7c-5p, Exo-miR-21-5p, and Exo-miR-30b-5p have been found as potential biomarkers for prognosing the severity and the adverse progression of renal injury in type 2 DN. Urinary Exo-miR-451-5p shows the potential usefulness as a sensitive prognostic biomarker, instead of albumin excretion levels, to serially monitor the early renal damage in diabetes. On the other hand, the expression of a panel of eight serum Exo-miRs (miR- 1246, miR-642a-3p, let-7c-5p, miR-1255b-5p, let-7i-3p, miR-5010-5p, miR-150-3p, and miR-4449) is DN-specific and provides the promising biomarker candidate for the diagnosis of DN. Further, Exo-miR-29 was found as a potential serum-based biomarker for the early diagnosis of DN in pediatric T1DM patients.

The aforementioned conclusions of the present review article indicate that Exo-miRs donate great promise for the future progression in DN treatments, permitting more patients suffering from DN to benefit, more likely in the near future.

## Abbreviations

DN: Diabetic nephropathy
ESRD: End-stage renal disease
DM: Diabetes, mellitus
T1DM: Type I DM
T2DM: Type II DM
ECM: Extracellular matrix
GBM: Glomerular basement membrane
BUN: Blood urea nitrogen
eGFR: Estimated glomerular filtration rate
UACR: Urine albumin-to-creatinine ratio
RAAS: Renin–angiotensin–aldosterone system
GLP-1: Glucagon-like peptide-1
SGLT2: Sodium-glucose co-transporter 2
miRs: MicroRNAs
Exo-miRs: Exosomal miRs
adMSCs: Adipose-derived mesenchymal stem cells
ADSCs: Adipose-derived stem cells
hUSCs: Human urine‐derived stem cells
BMSCs: Bone marrow mesenchymal stem cells
GMCs: Glomerular mesangial cells
HG: High glucose
IL-6: Interleukin-6
HDAC1: Histone deacetylase 1
ET-1: Endothelin-1
GFB: Glomerular filtration barrier
mTOR: Mechanistic target of rapamycin
EMT: Epithelial-mesenchymal transition
ZEB2: Zinc finger E-box-binding homeobox-2
VEGFA: Vascular endothelial growth factor A
USP22: Ubiquitin-specific peptidase 22
TGF-β: Transforming growth factor beta
MAPK: Mitogen-activated protein kinase
AP-1: Activator protein-1
NF-κB: Nuclear factor-κB
BMI: Body mass index
